# Effect of valproate and lithium on dementia onset risk in bipolar disorder patients

**DOI:** 10.1038/s41598-022-18350-1

**Published:** 2022-08-19

**Authors:** Woori Moon, Eunjeong Ji, Juyoung Shin, Jun Soo Kwon, Ki Woong Kim

**Affiliations:** 1 Department of Clinical Medical Science, Seoul National University College of Medicine, Seoul, Korea; 2 Department of Neuropsychiatry, Seoul National University Bundang Hospital, 82 Gumi-ro 173beon-gil, Bundang-gu, Seongnam, 13620 Korea; 3 Medical Research Collaborating Center (MRCC), Seoul National University Bundang Hospital, Seongnam, Korea; 4 School of Pharmacy, Sungkyunkwan University, Suwon, Korea; 5 Department of Neuropsychiatry, Seoul National University Hospital, 103 Daehak-ro, Jongno-gu, Seoul, 03080 Korea; 6 Department of Brain and Cognitive Science, Seoul National University College of Natural Sciences, Seoul, Korea; 7 Department of Psychiatry and Behavioral Science, Seoul National University College of Medicine, Seoul, Korea

**Keywords:** Neuroscience, Psychology, Diseases, Neurology

## Abstract

Although valproate and lithium are most commonly prescribed for bipolar disorder patients, studies comparing their effects on the risk of dementia are limited. Choosing a safer mood stabilizer is clinically crucial as elderly bipolar disorder patients are at high risk of dementia onset. Therefore, we aim to evaluate and compare the effects of valproate and lithium on the risk of dementia in elderly bipolar disorder patients. This study involved 4784 bipolar disorder patients aged 50 years or older from the Korean Health Insurance Review and Assessment Service database. We estimated the risk of dementia in valproate-only users, lithium-only users, and both users compared to both medication non-users using multivariable Cox proportional hazard models. Compared to non-users, valproate-only users and both users showed a higher risk of dementia (59% and 62%, respectively). In sub-group analysis, valproate increased the dementia risk when prescribed for at least 59 days or 23 cumulative defined daily doses. However, the dementia risk associated with lithium is unclear. Therefore, we concluded that lithium has the potential to be the safer choice as a mood stabilizer over valproate for elderly bipolar disorder patients considering the risk of dementia.

## Introduction

Older adults aged 60 years and over account for 25% of all bipolar disorder patients^1^. As affective temperament in bipolar disorder patients is associated with lifetime suicide attempts or risky bereavement, continuous lifetime monitoring and treatment are necessary for old age patients^2,3^. Bipolar disorder is associated with an increased risk of dementia due to various factors, including treatment using psychiatric medications^4–6^. As valproate and lithium are the most frequently prescribed medications for bipolar disorder in older adults^7^, estimation of dementia onset risk associated with valproate or lithium is clinically crucial.

In preclinical studies, lithium was found to induce proliferation and specification of neurons by inhibiting glycogen synthase kinase 3 (GSK-3β)^8–12^ while the effects of valproate on neurons are controversial^13–15^. Studies that directly investigated the causal effects of valproate and lithium on the risk of dementia in patients with bipolar disorder are very limited and subjected to methodological limitations^16,17^. A population-based retrospective cohort study from Taiwan reported that valproate increased the risk of dementia in bipolar disorder patients^18^. Two population-based, retrospective cohort studies from Denmark and the United States reported that lithium reduced the risk of dementia in bipolar disorder patients^16,17^. Although aforementioned studies adjusted anticonvulsant^16,17^ or mood stabilizer^16,18^ treatment, none of those excluded valproate-treated patients from their lithium-nontreated control groups^16,17^ and vice versa^18^. These results, therefore, do not necessarily indicate that valproate or lithium may change the risk of dementia in patients with bipolar disorder, but may simply indicate that the risk of dementia may be different between valproate-treated and lithium-treated bipolar disorder patients. In addition, none of these studies directly compared the risk of dementia between valproate users and lithium users with common matched non-users. Thus, it is difficult to determine whether it is valproate or lithium that is less or more associated with the risk of dementia in bipolar patients.

In this context, we hypothesized that lithium would be less associated with risk of dementia onset than valproate for older age bipolar disorder patients. To verify this hypothesis, we compared the risk of dementia onset in patients who were prescribed valproate only (VALP), lithium only (LITH), or both valproate and lithium (BOTH) to common matched controls who were neither prescribed valproate nor lithium (NONE) after controlling for potential confounding factors. In this study, we used Korean national health insurance claim data of bipolar disorder patients aged 50 years or older.

## Results

### Characteristics of the participants

**Figure 1:**
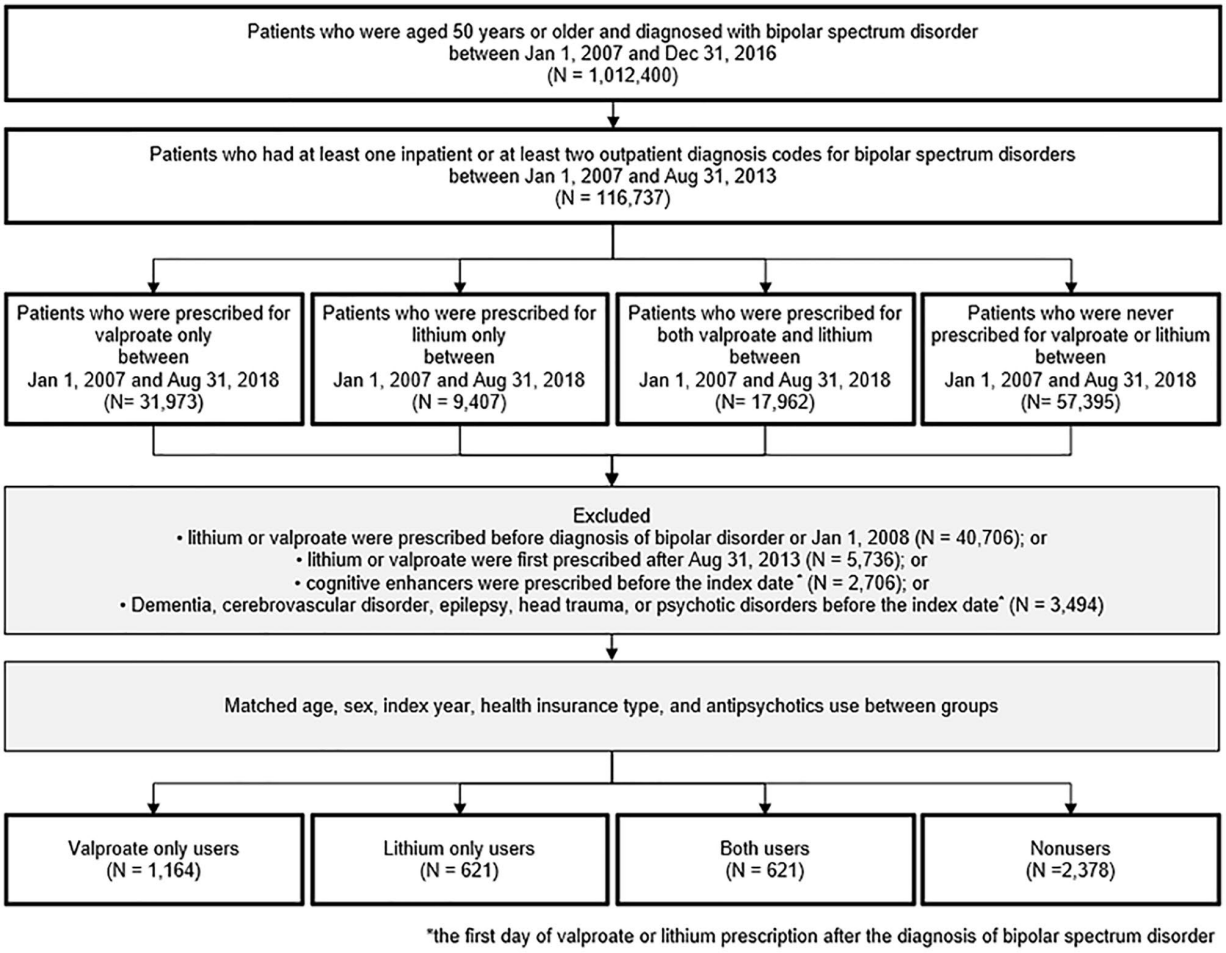
Assembly of study cohort with valproate only users, lithium only users, both valproate and lithium users, and nonusers.

The study cohort comprised of 1164 VALP group patients, 621 LITH group patients, 621 BOTH group patients, and 2378 NONE group patients. Figure 1 summarizes the cohort assembly process. The mean follow-up durations in all four groups were over 6.6 years. As summarized in Table 1, the average age of each group was around 58 years old, and females were more dominant in all groups. Most covariates were well balanced between four groups, with some exceptions in nonusers. Nonusers have more coronary artery disorder, depressive disorder and use more medications (Selective serotonin reuptake inhibitor (SSRI), Serotonin and norepinephrine reuptake inhibitors (SNRI), Tricyclic antidepressants (TCA), benzodiazepine, anti-inflammatory agents, narcotics, H2 receptor antagonist, statin, platelet aggregation inhibitor, antihypertensive, and flourquinolones) than medication users. As we matched the use of antipsychotics, the prescription rates of antipsychotics did not differ significantly between the four groups. Table 1 shows detailed demographic and clinical characteristics of the four groups.

**Table 1:** Baseline characteristics of the valproate only users, lithium only users, both valproate and lithium users, and nonusers.

	VALP	LITH	BOTH	NONE	*p*	Post-hoc*
	(n = 1164)	(n = 621)	(n = 621)	(n = 2378)		
Age (y)	57.92 ± 6.82	58.08 ± 6.95	58.08 ± 6.95	58.05 ± 6.93	0.94	
50–60	780 (67.01)	410 (66.02)	410 (66.02)	1574 (66.19)	0.99	
60–70	292 (25.09)	157 (25.28)	157 (25.28)	597 (25.11)		
70–80	92 (7.90)	54 (8.70)	54 (8.70)	207 (8.70)		
> 80	0 (0.00)	0 (0.00)	0 (0.00)	0 (0.00)		
Sex					0.99	
Male	447 (38.40)	237 (38.16)	237 (38.16)	898 (37.76)		
Female	717 (61.60)	384 (61.84)	384 (61.84)	1480 (62.24)		
**Type of insurance**						
Health insurance	1031 (88.57)	545 (87.76)	545 (87.76)	2177 (91.55)		a, b, c < d
Medical aid	133 (11.43)	76 (12.24)	76 (12.24)	201 (8.45)		
**CCI score**					< 0.01	
≤ 1	346 (55.72)	634 (54.47)	359 (57.81)	1155 (48.57)		a, b, c > d
2	123 (19.81)	210 (18.04)	107 (17.23)	464 (19.51)		
≥ 3	152 (24.48)	320 (27.49)	155 (24.96)	759 (31.92)		
**Comorbidities**						
Hypertension	28 (2.41)	9 (1.45)	13 (2.09)	57 (5.40)	0.52	
Atrial fibrillation	9 (0.77)	4 (0.64)	3 (0.48)	10 (0.42)	0.53	
Coronary artery disorder	74 (6.36)	25 (4.03)	29 (4.67)	189 (7.95)	< 0.01	a, c < d
Peripheral vascular disease	19 (1.63)	9 (1.45)	8 (1.29)	33 (1.39)	0.93	
Diabetes	175 (15.03)	94 (15.14)	86 (13.85)	386 (16.23)	0.47	
Hyperlipidemia	96 (8.25)	47 (7.57)	49 (7.89)	235 (9.88)	0.14	
**Psychotic disorder**						
Depressive disorder	475 (40.81)	261 (42.03)	241 (38.81)	1328 (55.85)	< 0.01	a, b, c < d
Substance related disorder	9 (0.77)	1 (0.16)	3 (0.48)	11 (0.46)	0.38	
Alcohol related disorder	168 (14.43)	60 (9.66)	51 (8.21)	159 (6.69)	< 0.01	b > a > d
**Medication**						
Anticholinergics	523 (44.93)	294 (47.34)	305 (49.11)	1034 (43.48)	0.05	
Antipsychotics	935 (80.33)	483 (77.78)	483 (77.78)	1862 (78.30)	0.44	
SSRI/SNRI	607 (52.15)	310 (49.92)	284 (45.73)	1491 (62.70)	< 0.01	a, b, c < d
Antiepileptics	277 (23.80)	141 (22.71)	126 (20.29)	548 (23.04)	0.40	
TCA	432 (37.11)	228 (36.71)	211 (33.98)	1084 (45.58)	< 0.01	a, b, c < d
Benzodiazepine	1064 (91.41)	551 (88.73)	567 (91.30)	2209 (92.89)	< 0.01	a < d
Anti-inflammatory analgesics	1002 (86.08)	515 (82.93)	518 (83.41)	2146 (90.24)	< 0.01	a, b, c < d
Narcotic analgesics	786 (67.53)	383 (61.67)	395 (63.61)	1708 (71.83)	< 0.01	a, b, c < d
H2RA	862 (74.05)	430 (69.24)	446 (71.82)	1886 (79.31)	< 0.01	a, b, c < d
ERT	100 (8.59)	48 (7.73)	49 (7.89)	215 (9.04)	0.66	
Antidiabetic agents	191 (16.41)	87 (14.01)	97 (15.62)	402 (16.90)	0.36	
Statin	297 (25.52)	141 (22.71)	152 (24.48)	710 (29.86)	< 0.01	a, b, c < d
Anticoagulant	206 (17.70)	95 (15.30)	105 (16.91)	413 (17.37)	0.60	
Platelet aggregation inhibitors	337 (28.95)	137 (22.06)	160 (25.76)	799 (33.60)	< 0.01	a, b, c < d
Antihypertensive	674 (57.90)	336 (58.94)	358 (57.65)	1533 (64.47)	< 0.01	b, c < d
Fluorquinolones	413 (35.48)	205 (33.01)	202 (32.53)	995 (41.84)	< 0.01	a, b, c < d
Others**	347 (29.81)	188 (30.27)	177 (28.50)	960 (40.37)	< 0.01	
Mean follow-up duration (y)	6.64 ± 2.80	6.87 ± 2.66	6.89 ± 2.62	7.01 ± 2.51	< 0.01	b < d
**Index year**					0.72	
2008	187 (16.07)	103 (16.59)	103 (16.59)	448 (18.84)		
2009	300 (25.77)	165 (26.57)	165 (26.57)	541 (22.75)		
2010	177 (15.21)	94 (15.14)	94 (15.14)	350 (14.72)		
2011	204 (17.53)	106 (17.07)	106 (17.07)	417 (17.54)		
2012	210 (18.04)	107 (17.23)	107 (17.23)	433 (18.21)		
2013**	86 (7.39)	46 (7.41)	46 (7.41)	189 (7.95)		

Values are presented as mean ± standard deviation, number only, or number (%).

py, person-years; SSRI, selective serotonin receptor inhibitor; SNRI, serotonin norepinephrine receptor inhibitor; TCA, ticyclic antidepressants; H2RA, histamine 2 receptor antagonist; ERT, estrogen replacement therapy.

*a: Lithium only users, b: Valproic acid only user, c: both users, d: both non-users.

**Others included bicalutamide, buspirone, digoxinm, and tirpramide.

***Until August.

### Hazard ratio analysis for dementia onset

In order to select covariates for adjustment, we ran univariable Cox proportional hazard analysis for the factors listed Table 1. As a result, diabetes (HR 1.43, 95% CI 1.03–1.99), alcohol-related disorder (HR 1.98, 95% CI 1.13–3.48) and use of antiepileptics (HR 1.42, 95% CI 1.05–1.93) were selected as covariates that should be adjusted in the analysis on the associations of valproate and/or lithium with the risk of dementia (eTable 1 in the Supplement).

In a multivariable Cox proportional hazard analysis, the VALP group (HR 1.56, 95% CI 1.23–1.97) and the BOTH group (HR 1.62, 95% CI 1.23–2.13) but not LITH group (HR 1.24, CI 0.92–1.67) were at higher risk of dementia compared to the NONE group (Table 2). In sensitivity analysis which defines dementia cases not only by disease code but also by the prescription of cognitive enhancers (donepezil, memantine, rivastigmine and galantamine) for at least 7 days, we found similar results. The VALP group (HR 1.70, 95% CI 1.22–2.37) and the BOTH group (HR 1.51, 95% CI 1.02–2.23) but not LITH group (HR 1.43, 95% CI 0.96–2.14) were at higher risk of dementia compared to the NONE group (eTable 2 in the Supplement).

**Table 2:** Risk of dementia in valproate only users, lithium only users, and both valproate and lithium users compared to nonusers.

	n	Event	py	Unadjusted*	*P* value	Adjusted*^,†^	*P* value
Valproate only users	1164	130	7728	1.59 (1.26–2.02)	< 0.01	1.56 (1.23–1.97)	< 0.01
Lithium only users	621	65	4265	1.26 (0.94–1.69)	0.13	1.24 (0.92–1.67)	0.15
Both users	621	80	4279	1.62 (1.23–2.13)	< 0.01	1.62 (1.23–2.13)	< 0.01
Nonusers	2378	185	16,660	Ref		Ref	

py, person-years.

*Hazard ratio for dementia with 95% confidence intervals estimated by multivariable Cox proportional hazard models.

^†^Adjusted for diabetes, alcohol-related disorder, and use of antiepileptics.

### Dose–response analysis

We analyzed the risk of dementia according to the cumulative defined daily dose (DDD), cumulative prescription duration (CPD) (Table 3). In the VALP group, the risk of dementia was significantly higher in the middle and high DDD (middle DDD group: HR 1.98, 95% CI 1.34–2.89; high DDD group: HR 1.59, 95% CI 1.12–2.27) or middle and long CPD groups (middle CPD group: HR 2.11, 95% CI 1.45–3.07; long CPD group: HR 1.60, 95% CI 1.13–2.26) but not in the low DDD (HR 1.25, 95% CI 0.84–1.87) or short CPD group (HR 1.15, 95% CI 0.76–1.74) compared to the NONE group. However, exposure to lithium did not show statistically significant association with dementia risk in any tertile group by DDD or CPD (Table 3).

**Table 3:** Risk of dementia in valproate only users and lithium only users compared to nonusers according to the cumulative dose and days of prescription.

	N(event)	Unadjusted*	*P* value	Adjusted*^,†^	*P* value
***By cumulative dose (DDD***†***)***					
**Valproate only users**					
Low (< 23)	383 (34)	1.31 (0.88–1.96)	0.18	1.25 (0.84–1.87)	0.28
Mid (23–107)	396 (44)	2.03 (1.40–2.95)	< 0.01	1.98 (1.34–2.89)	< 0.01
high (≥ 107)	385 (52)	1.62 (1.14–2.31)	< 0.01	1.59 (1.12–2.27)	< 0.01
**Lithium only users**					
Low (< 140)	206 (21)	1.34 (0.82–2.21)	0.24	1.31 (0.79–2.17)	0.29
Mid (140–665)	211 (24)	1.26 (0.79–2.01)	0.33	1.20 (0.75–1.93)	0.45
High (≥ 665)	204 (20)	1.10 (0.65–1.87)	0.72	1.12 (0.66–1.91)	0.67
***By cumulative dose (in gram)***					
**Valproate only users**					
Low (< 35)	383 (34)	1.31 (0.88–1.96)	0.18	1.25 (0.84–1.87)	0.28
Mid (35–161)	396 (44)	2.03 (1.40–2.95)	< 0.01	1.98 (1.34–2.89)	< 0.01
High (≥ 161)	385 (52)	1.62 (1.14–2.31)	< 0.01	1.59 (1.12–2.27)	< 0.01
**Lithium only users**					
Low (< 23)	206 (21)	1.34 (0.82–2.21)	0.24	1.31 (0.79–2.17)	0.29
Mid (23–111)	211 (24)	1.26 (0.79–2.01)	0.33	1.20 (0.75–1.93)	0.45
High (≥ 111)	204 (20)	1.10 (0.65–1.87)	0.72	1.12 (0.66–1.91)	0.67
***By days of prescription (days)***					
**Valproate only users**					
Short (< 59)	380 (31)	1.21 (0.80–1.82)	0.37	1.15 (0.76–1.74)	0.52
Mid (59–244)	398 (46)	2.18 (1.50–3.15)	< 0.01	2.11 (1.45–3.07)	< 0.01
Long (≥ 244)	386 (53)	1.63 (1.15–2.30)	< 0.01	1.60 (1.13–2.26)	< 0.01
**Lithium only users**					
Short (< 42)	203 (18)	1.19 (0.70–2.03)	0.51	1.14 (0.67–1.96)	0.63
Mid (42–183)	213 (23)	1.20 (0.75–1.92)	0.46	1.14 (0.71–1.85)	0.58
Long (≥ 183)	205 (24)	1.31 (0.80–2.14)	0.28	1.33 (0.81–2.18)	0.26

*Hazard ratio with 95% confidence intervals compared to the nonusers (number = 2378, event = 185).

^†^Adjusted for diabetes, alcohol related disorder, and use of antiepileptics.

^†^DDD = defined daily dose, 1 DDD = 1.5 g for valproate, 24 mmol for lithium.

## Discussion

To the extent of the author’s knowledge, this is the first study that directly compares the risk of dementia between valproate users, lithium users, and both users compared with the matched non-user controls among elderly patients with bipolar disorder. We found that patients who were prescribed only valproate or both valproate and lithium showed a 56% or 62% increased risk of dementia, respectively than those who were not prescribed both medications. However, lithium-only users did not show a significant increase in the risk of dementia. Our result indicates that lithium has the potential to be a relatively safer choice than valproate when considering the risk of dementia in elderly bipolar disorder patients. Given the risk of valproate-associated dementia, we recommend regularly monitoring cognitive function in elderly bipolar disorder patients using valproate.

The results of this study support the dementia onset risk associated with valproate shown in previous studies. In previous studies, valproate reduced cell proliferation at therapeutic plasma levels^19^ and impaired cognitive function in bipolar disorder patients^20^. Prior to this study, only one study from Taiwan used real-world patient data to investigate the effect of valproate on the risk of dementia in older patients with bipolar disorder at population level. The Taiwanese study reported that valproate-treated patients had a 73% higher risk of dementia than valproate-untreated patients^18^, which is consistent with the findings of the current study. In addition, the current study further discovered that the risk of dementia increased significantly when valproate was prescribed for at least about two months or 23 DDD. To the best of our knowledge, no study has investigated the dose–response relationship between valproate prescription and the risk of dementia in bipolar disorder patients. Our finding indicates that regular monitoring of cognitive function would be helpful for older aged bipolar patients prescribed valproate for approximately two months or longer.

Unlike valproate, based on our result, it is unclear whether lithium increases the risk of dementia or not. However, our finding does not support the lithium’s protective effect against dementia which was found in some previous studies. Prior to this research, two retrospective, population-based cohort studies using health insurance claims data from Denmark and the United States had investigated the effects of lithium on the risk of dementia in bipolar patients^16,17^. More than four prescriptions of lithium reduced the risk of dementia in the Danish study^16^, and prescriptions of lithium for 10–12 months reduced the risk of dementia in the U.S. study^17^. Unlike current study, the U.S. or Danish study did not exclude valproate users from non-user control group. Valproate was found to increase the risk of dementia in both the current study and the aforementioned Taiwanese study^18^. Therefore, to precisely estimate the effect of lithium on dementia, valproate users should be excluded or at least matched between lithium users and non-users. Other studies reported mixed results regarding the effects of lithium on human cognitive function. In randomized controlled trials on patients with mild cognitive impairment^21,22^ or Alzheimer’s disease^23^ without affective disorders, sub-therapeutic treatment with lithium (serum levels below 0.5 mEq/L) for one to three years improved cognitive performance^21,22^, delayed cognitive decline^23^, increased amyloid-beta peptide^22^ and decreased phosphorylated tau^21^ in the cerebrospinal fluid. However, in a population-based, nested case–control study using data from the Taiwan National Health Insurance Program, lithium treatment was not associated with the risk of Alzheimer's disease in bipolar patients^24^. A meta-analysis of six studies (two case–control studies and four pre-post comparison studies) reported that therapeutic lithium treatment (serum level: 0.7–1.2 mEq/L) for approximately four years impaired immediate verbal learning, memory, and psychomotor performance of affective disorder patients, and long-term lithium treatment was associated with greater impairment in psychomotor performance^25^. Combining the results of current and past studies, more long-term, real-world data is required to clarify the effect of lithium on the onset of dementia in clinical settings.

Nevertheless, the results of our study suggest that lithium would be a safer option compared to valproate regarding the risk of dementia in elderly bipolar disorder patients. Considering that bipolar patients are predisposed to developing dementia^4–6^, our findings provide meaningful information to support the clinical choice of mood stabilizers for elderly patients.

There are several limitations to be considered. As we use bipolar disorder patients who have not been prescribed both valproate and lithium as the reference group, cofounding by indication should be considered as limitation. The major concern regarding confounding by indication is the difference in bipolar disorder severity between non-user controls and valproate or lithium users. One could argue that the higher risk of dementia in the VALP group could be biased from greater bipolar disorder severity in the VALP group than NONE group. However, such difference in disease severity could also exist between the NONE and LITH group. Nevertheless, the LITH group did not show increased risk of dementia in current study. Such differences in dementia risk patterns between the matched VALP and LITH groups partially reduce the concern of confounding by indication. In addition, to alleviate confounding by indication, we balanced the clinical characteristics between groups by matching age, sex, index year, and health insurance type and the use of antipsychotics, as they are frequently prescribed for bipolar disorder along with valproate or lithium^16,26–28^. Even still, the unadjusted effect of disease severity could have remained. Therefore, we expect further investigation overcoming such intrinsic limitations of claim data. This study analyzed national insurance claim data from Health Insurance Review and Assessment Service (HIRA). HIRA data provides valuable information based on real-world, but it also has several limitations. First, key risk factors of dementia, such as the level of education and apolipoprotein E genotype were not controlled as they were not available in the HIRA data. Second, the record of dementia code in claim data does not necessarily indicate clinical diagnosis of dementia. Notably, we could not specify the specialty of a physician who made the diagnosis due to the limitation of data availability. To ensure the validity of diagnosis, we conducted sensitivity analysis which defines dementia cases not only by disease code but also by the prescription of cognitive enhancers for at least 7 days, and found consistent results (eTable 2 in the Supplement). Third, due to concerns regarding the accuracy of medical records of dementia subtypes, we analyzed the effects of valproate and lithium on all-cause dementia. Finally, although we ensured that there was at least a one-year wash-out period for valproate and lithium, lifetime exposure to lithium and valproate might have also influenced the risk of dementia.

In conclusion, our study found that prolonged use of valproate could increase the risk of dementia, whereas the risk associated with lithium is unclear in elderly bipolar patients. Regarding the susceptibility to the onset of dementia in bipolar disorder patients, lithium has the potential to be a safer choice than valproate as a mood stabilizer in elderly patients. In the case prescription of valproate is unavoidable, regular monitoring of cognitive function is necessary for the timely detection of cognitive decline.

## Methods

### Data source and study cohort

We obtained the dataset from the Korean HIRA database between January 1, 2007 and August 31, 2018. The HIRA database comprises of ~ 59 million individuals and is representative of almost all Koreans insured by the National Health Insurance and National Medicaid. The HIRA database contains data on patient demographics, diagnosis codes, prescription records, medical procedures and services, types of health care institutions, medication utilization, and admission dates.

From the HIRA database, we identified 116,737 registrants who were 50 years or older and had either at least one inpatient claim or at least two outpatient claims with ICD-10 codes of bipolar spectrum disorders (F30, F31, and F34.0) as their primary or secondary diagnosis between January 1, 2007 and August 31, 2013.

Among them, we identified patients who received at least one prescription for valproate between January 1, 2007, and August 31, 2018, but did not receive lithium as valproate only users and vice versa for lithium only users (N = 31,973 for VALP group, N = 9.407 for LITH group). We also found patients who prescribed both valproate and lithium at least one time for each between January 1, 2007, and August 31, 2018 (N = 17,692 for BOTH group).

From these patients, we excluded individuals who: (1) were prescribed valproate and/or lithium before obtaining a diagnosis code for bipolar spectrum disorder; (2) had a diagnosis code for psychotic disorders, dementia, cerebrovascular disorder, Parkinson disorder, epilepsy, or traumatic head injury as the primary diagnosis before being prescribed valproate and/or lithium; or (3) were prescribed acetylcholinesterase inhibitors or memantine before being prescribed valproate and/or lithium. We further excluded individuals who were prescribed valproate and/or lithium between January 1, 2007 and December 31, 2007 to ensure that there was at least a one-year wash-out period in patients who were prescribed valproate or lithium before 2008. As we have data until august 31, 2018, in order to secure at least 5-year follow up data, we excluded patients who first prescribed valproate or lithium after august 31, 2013. (Fig. 1).

After that, we matched age, sex, index year, and health insurance type between VALP group, LITH group, BOTH group, and patients who were never prescribed valproate or lithium between Jan 1, 2017, to Aug 31, 2018, as reference group (NONE group). In order to minimize the confounding by indication, we also matched the use of antipsychotics between four groups. Finally, 1164 VALP group, 621 LITH group, 621 BOTH group and 2378 NONE group patients are included in the analysis.

**Figure 2:**
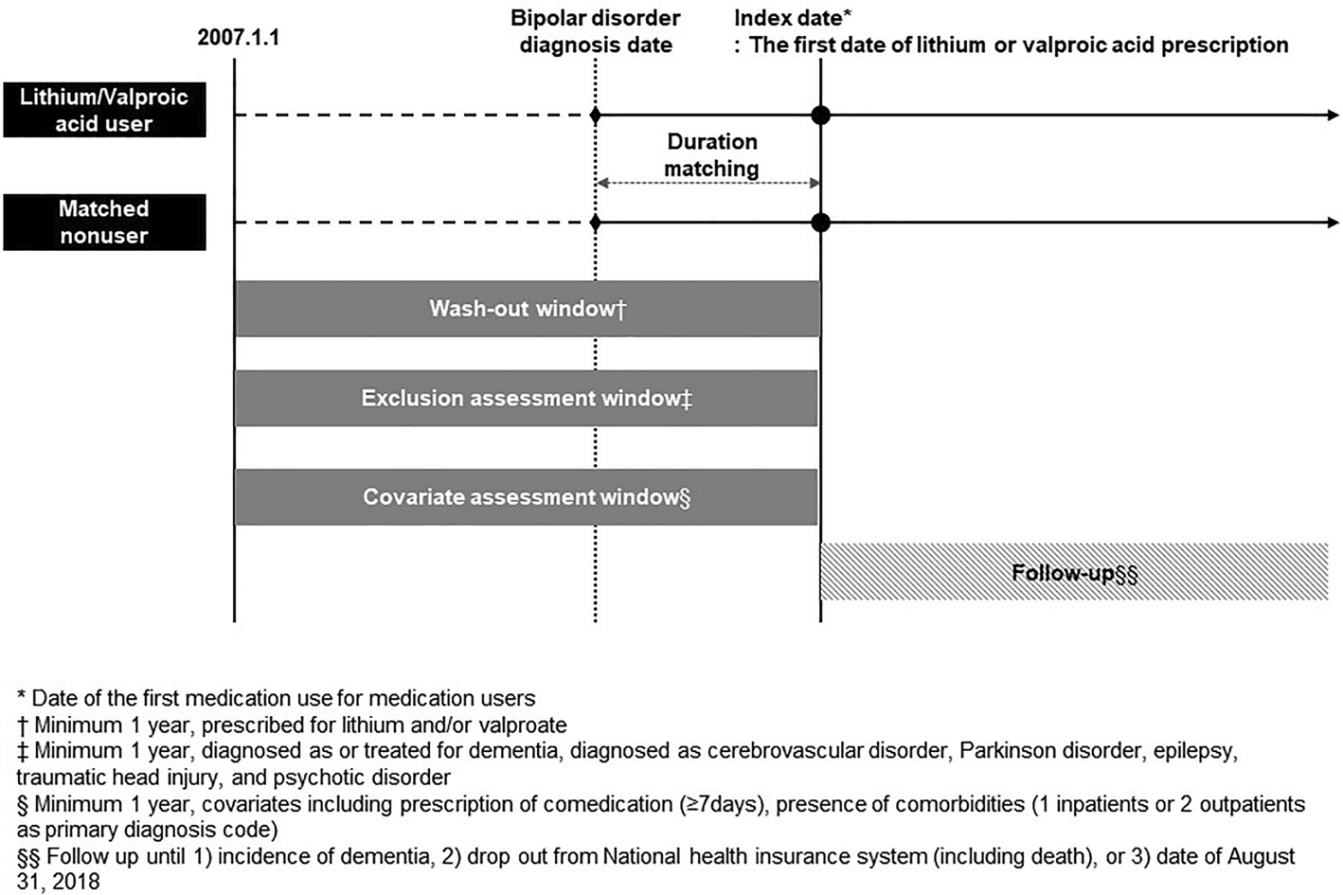
Description of study design.

We defined the index date as the first day of valproate or lithium prescription after the diagnosis of bipolar spectrum disorder in the VALP, LITH, and BOTH groups. In the NONE group, we defined the index date by adding the interval between the date of bipolar disorder diagnosis and the index date of the matched VALP, LITH, and BOTH groups to the date of bipolar disorder diagnosis of the NONE group (Fig. 2).

This study was approved by the Institutional Review Board of the Seoul National University Bundang Hospital (IRB no. X-1906-546-901), and conformed to the ethical guidelines of the World Medical Association Declaration of Helsinki. Informed consents were waived from the Institutional Review Board of the Seoul National University Bundang Hospital due to the retrospective design.

### Exposures

Exposure to valproate (ATC; N03AG01) or lithium (ATC; N05AN01) was evaluated between the index date and the last day of follow-up. To assess dose–response relationship, we calculated DDD, CPD of valproate and lithium in days. When calculating DDD, we adapted the widely used drug standardization method developed by the World Health Organization^21^. We divided the level of exposure into tertiles of cumulative DDD and CPD.

### Outcome

The outcome of the study was the incident diagnosis of dementia. We defined incident dementia as having at least one inpatient claim or at least two outpatient claims with ICD-10 codes of dementia (F00, F01, F02, F03, G30, G31.0, G31.8, or G31.9) as the *primary* diagnosis at least one year after the index date. We did not include the patients whose dementia diagnosis code occurred within a year from index date as incident dementia to avoid protopathic bias due to preexisting undiagnosed dementia and to address disease latency for drug-induced dementia. We followed the patients from the index date until one of the following conditions were met: (1) the diagnosis of dementia; (2) the patient was excluded from the HIRA database due to reasons including death; or (3) the date of August 31, 2018.

### Confounding factors

We defined the following factors as potential confounders: (1) the existence of comorbidities, evaluated as having at least one inpatient or at least two outpatient ICD-10 codes of comorbidities (Table 1) before the index date; and (2) the use of prescribed comedications (Table 1) for at least one week before the index date. Using the comorbidities, we estimated the Charlson Comorbidity Index (CCI) score^29^.

### Statistical analyses

We compared the risk of dementia in the VALP, LITH, and BOTH groups to that of the NONE group using a multivariable Cox proportional hazard model. In this analysis, we adjusted the results for confounding factors that showed a significant association with the risk of dementia in the univariable Cox proportional hazard models (eTable 1 in the supplement).

We subsequently categorized the DDD and CPD of valproate and lithium into tertiles, estimated the risk of dementia in each tertile using the multivariable Cox proportional hazard models, and adjusted the results for the aforementioned confounding factors.

We also performed similar analyses after modifying the definition of incident dementia as the patients with dementia code in primary code and prescription of cognitive enhancer for 7 days or longer. All statistical analyses were performed using SAS Enterprise 7.1 for Windows (SAS Institute Inc., Cary, NC, USA), and regarded two-tailed *p* values below 0.05 as statistically significant.

### Ethics declarations

This study was approved by the Institutional Review Board of the Seoul National University Bundang Hospital (IRB no. X-1906-546-901), and conformed to the ethical guidelines of the World Medical Association Declaration of Helsinki.

### Consent for participate

Informed consents were waived from the Institutional Review Board of the Seoul National University Bundang Hospital due to the retrospective design.

## Supplementary Information

Supplementary Information.

## Data Availability

Data available on reasonable request from the authors.
