# Supplementary material for: Effect of valproate and lithium on dementia onset risk in bipolar disorder patients
Source: Sci Rep. 2022 Aug 19;12:14142. doi: 10.1038/s41598-022-18350-1 (PMC9391483; doi:10.1038/s41598-022-18350-1)
Supplement: Supplementary file 1 — Supplementary Information. [file 41598_2022_18350_MOESM1_ESM.docx]

**Supplementary information**

**Effect of valproate and lithium on dementia onset risk** **in bipolar disorder patients**

Woori Moon, Ph.D., Eunjeong Ji, Ph.D., Juyoung Shin, Ph.D., Jun Soo Kwon, Ph.D., and Kiwoong Kim, Ph.D.

**Table of Contents**

1. **eTable in the Supplement ………………………….………….……2**
2. **eTable in the Supplement ………………………….………….……4**

**eTable 1. Risk of dementia associated with potential confounding factors in the study cohort with valproate only users, lithium only users, both valproate and lithium users, and nonusers**

| **Variable** | **Hazard ratio ^†^** | **p** |
| --- | --- | --- |
| **Number of admissions** | 1·01 (0·96–1·05) | 0·84 |
| **CCI score** |  |  |
| **≤ 1** | Reference |  |
| **2** | 1·01 (0·71–1·45) | 0·94 |
| **≥ 3** | 1·21 (0·90–1·64) | 0·21 |
| **Comorbidities** |  |  |
| **Hypertension** | 1·12 (0·45–2·80) | 0·81 |
| **Atrial fibrillation** | 1·14 (0·23–5·72) | 0·87 |
| **Coronary artery disorder** | 0·56 (0·30–1·05) | 0·07 |
| **Peripheral vascular disease** | 2·18 (0·99–4·81) | 0·05 |
| **Diabetes^‡^** | 1·43 (1·03–1·99) | 0·03 |
| **Hyperlipidemia** | 0·89 (0·54–1·46) | 0·63 |
| **Depressive disorder** | 1·16 (0·87–1·55) | 0·32 |
| **Substance related disorder** | 2·38 (0·58–9·79) | 0·23 |
| **Alcohol related disorder^‡^** | 1·98 (1·13–3·48) | 0·02 |
| **Medication** |  |  |
| **Anticholinergics** | 1·16 (0·88–1·52) | 0·29 |
| **SSRI/SNRI** | 1·02 (0·75–1·38) | 0·92 |
| **Antiepileptics^‡^** | 1·42 (1·05–1·93) | 0·02 |
| **TCA** | 1·09 (0·83–1·44) | 0·52 |
| **Benzodiazepine** | 0·96 (0·59–1·56) | 0·87 |
| **Anti-inflammatory analgesics** | 0·87 (0·58–1·32) | 0·52 |
| **Narcotic analgesics** | 0·89 (0·66–1·21) | 0·46 |
| **H2RA** | 0·95 (0·69–1·31) | 0·76 |
| **ERT** | 0·65 (0·32–1·33) | 0·24 |
| **Antidiabetic agents** | 1·26 (0·91–1·74) | 0·17 |
| **Statin** | 0·85 (0·62–1·16) | 0·31 |
| **Anticoagulant** | 0·98 (0·69–1·37) | 0·89 |
| **Platelet aggregation inhibitors** | 0·95 (0·72–1·26) | 0·73 |
| **Antihypertensive** | 1·07 (0·80–1·43) | 0·64 |
| **Fluorquinolones** | 0·92 (0·69–1·23) | 0·57 |
| **Others^§^** | 1·16 (0·87–1·54) | 0·31 |

CCI: Charlson comorbidity index; SSRI: selective serotonin receptor inhibitor; SNRI: serotonin norepinephrine receptor inhibitor; TCA: ticyclic antidepressants; H2RA: histamine 2 receptor antagonists; ERT: estrogen replacement therapy.

^†^ Hazard ratio for dementia with 95% confidence interval in the univariable Cox proportional hazards model, patients without admission are used as reference in Number of admission category, patients with CCI score ≤ 1 are used as reference in CCI score category, patients without each of comorbidity are used as reference in comorbidities category, each of medication nonuser are used as reference in medication category.

^‡^Selected for adjustment in multivariable Cox proportional hazard model.

^§^included bicalutamide, buspirone, digoxinm, and tirpramide.

|  | N(event) | Unadjusted^*^ | P value | Adjusted^*,†^ | P value |
| --- | --- | --- | --- | --- | --- |
| ***By cumulative dose***  ***(DDD***^††^***)*** |  |  |  |  |  |
| VALP |  |  |  |  |  |
| low (<22) | 379 (21) | 1.23 (0.71 - 2.14) | 0.47 | 1.12 (0.64 - 1.96) | 0.70 |
| mid (22-103) | 393 (40) | 2.30 (1.39 - 3.80) | < 0.01 | 2.28 (1.37 - 3.79) | < 0.01 |
| high (≥103) | 382 (59) | 1.96 (1.27 - 3.04) | < 0.01 | 2.02 (1.30 - 3.13) | < 0.01 |
| LITH |  |  |  |  |  |
| low (<140) | 205 (22) | 1.93 (1.06 - 3.52) | 0.03 | 1.84 (1.00 - 3.38) | 0.05 |
| mid (140-722) | 208 (26) | 1.60 (0.89 - 2.86) | 0.11 | 1.55 (0.86 - 2.81) | 0.15 |
| high (≥722) | 204 (21) | 1.37 (0.70 - 2.65) | 0.36 | 1.46 (0.75 - 2.86) | 0.27 |
| ***By days of prescription***  ***(days)*** |  |  |  |  |  |
| VALP |  |  |  |  |  |
| short (<56) | 377 (33) | 1.22 (0.69 - 2.16) | 0.49 | 1.08 (0.61 - 1.93) | 0.79 |
| mid (56-226) | 394 (41) | 2.49 (1.52 - 4.08) | < 0.01 | 2.52 (1.53 - 4.14) | < 0.01 |
| long (≥226) | 383 (60) | 1.84 (1.19 - 2.84) | < 0.01 | 1.89 (1.22 - 2.92) | < 0.01 |
| LITH |  |  |  |  |  |
| short (<42) | 203 (21) | 1.84 (0.97 - 3.49) | 0.06 | 1.71 (0.90 - 3.27) | 0.10 |
| mid (42-205) | 210 (25) | 1.37 (0.76 - 2.47) | 0.29 | 1.33 (0.73 - 2.43) | 0.35 |
| long (≥205) | 204 (23) | 1.74 (0.94 - 3.22) | 0.08 | 1.86 (1.00 - 3.45) | 0.05 |

**eTable 2.** **Sensitivity analyses of risk of dementia in valproate only users, lithium only users, and both valproate and lithium users compared to nonusers with dementia ascertained by the primary diagnosis code and prescription of cognitive enhancer**

^*^Hazard ratio with 95% confidence intervals compared to the nonusers (number = 2,378, event = 100)

^†^Adjusted for diabetes, alcohol related disorder, and use of antiepileptics.

† DDD = defined daily dose, 1 DDD = 1.5g for valproate, 24mmol for lithium
